# Hippocampal State-Dependent Behavioral Reflex to an Identical Sensory Input in Rats

**DOI:** 10.1371/journal.pone.0112927

**Published:** 2014-11-14

**Authors:** Keita Tokuda, Michimasa Nishikawa, Shigenori Kawahara

**Affiliations:** 1 Department of Mathematical Informatics, Graduate School of Information Science and Technology, The University of Tokyo, 7-3-1 Hongo, Bunkyo-ku, Tokyo 113-8656, Japan; 2 Graduate School of Science and Engineering, University of Toyama, 3190 Gofuku, Toyama 930-8555, Japan; Federal University of Rio Grande do Norte, Brazil

## Abstract

We examined the local field potential of the hippocampus to monitor brain states during a conditional discrimination task, in order to elucidate the relationship between ongoing brain states and a conditioned motor reflex. Five 10-week-old Wistar/ST male rats underwent a serial feature positive conditional discrimination task in eyeblink conditioning using a preceding light stimulus as a conditional cue for reinforced trials. In this task, a 2-s light stimulus signaled that the following 350-ms tone (conditioned stimulus) was reinforced with a co-terminating 100-ms periorbital electrical shock. The interval between the end of conditional cue and the onset of the conditioned stimulus was 4±1 s. The conditioned stimulus was not reinforced when the light was not presented. Animals successfully utilized the light stimulus as a conditional cue to drive differential responses to the identical conditioned stimulus. We found that presentation of the conditional cue elicited hippocampal theta oscillations, which persisted during the interval of conditional cue and the conditioned stimulus. Moreover, expression of the conditioned response to the tone (conditioned stimulus) was correlated with the appearance of theta oscillations immediately before the conditioned stimulus. These data support hippocampal involvement in the network underlying a conditional discrimination task in eyeblink conditioning. They also suggest that the preceding hippocampal activity can determine information processing of the tone stimulus in the cerebellum and its associated circuits.

## Introduction

Coordinated interactions of lower-level sensorimotor systems with higher-level cognitive systems organize adaptive behaviors that are appropriate for the ongoing context. For example, changing the level of attention to a specific stimulus enables animals to respond quickly and accurately, but only when it is inferred by the context that the stimulus has behavioral saliency. This kind of attentional modulation, contextual dependence, or brain-state dependent information processing of sensory stimuli, is thought to be realized by the interaction between different systems in the brain, particularly by interactions between top-down and bottom-up processing [1]–[3] or between the internal brain-state and external stimuli [4]. However, the details and neurodynamics underlying these interactions between distributed systems remain to be elucidated.

Strong top-down modulation has also been reported even in the learning of a motor reflex, as is observed in classical eyeblink conditioning [5]. Classical eyeblink conditioning is one of the most extensively studied models for associative learning [6]–[8]. In this task, after exposure to pairs of a preceding neutral, conditioned stimulus (CS; such as a tone) and a behaviorally salient stimulus (unconditioned stimulus, US; such as a periorbital electrical shock), animals learn to blink in response to the CS. Cumulative evidence suggests that the underlying neural substrates for standard delay eyeblink conditioning involve the cerebellum and interconnected brainstem nuclei [7], [9], [10]. Conversely, many studies have reported significant involvements of higher-order neural circuits in the forebrain, such as the hippocampus and the medial prefrontal cortex [7], [11]–[13]. Because the basic neural circuit relevant for primary sensory processing and motor output has been well characterized, eyeblink conditioning provides a good model for studying the mechanism of attentional modulation realized by interactions between the higher-order and lower-order neural systems.

Some previous studies have reported strong top-down effects in eyeblink conditioning, even within the standard delay paradigm. Penick and Solomon showed that expression of the acquired conditioned responses (CRs) was disrupted when animals were placed in a different environment from that experienced during the acquisition phase [5]. However, this environmental contextual effect was ablated in animals subjected to pre-conditioning hippocampectomy. Poulos and colleagues went on to show that the expression of acquired CRs was substantially reduced with exposure to a different behavioral context between the acquisition and retention phases [14]. Together, these studies demonstrate that the underlying experimental settings have a strong contextual influence on the expression of acquired CRs. Rogers and Steinmetz developed a novel, contextually-based conditional discrimination task and showed that rabbits can acquire differential responses to identical CSs depending on chamber illumination, even though the transition between the contextual settings took place randomly in every trial [15]. Thus, they showed that the contextual modulation in eyeblink conditioning could also be flexible, as it can operate at a trial-by-trial time scale (of the order of tens of seconds). Another eyeblink conditioning paradigmthat is influenced by strong, top-down, contextual modulation on a trial-by-trial basis is a phenomenon known as conditional discrimination or occasion setting [16]. In this task, an identical CS is paired with a US only when the conditional cue (or “occasion setter”) is presented a few seconds before. Some studies using human subjects have suggested the involvement of the hippocampus in this conditional discrimination task [17]–[19]. While these previous studies clearly show a strong top-down modulation in eyeblink conditioning and suggest hippocampal involvement in the top-down modulation, little is currently known about the neural dynamics underlying the contextual dependency of eyeblink conditioning.

One of the candidates for the relevant neural dynamics that reflects a brain state of top-down modulation is the hippocampal theta rhythm. The hippocampus is the brain region for which we have the best understanding of the relationship between field potential oscillations and physiological function. The hippocampus has distinctive states characterized by the field potential oscillation, which have a strong correlation with ongoing behavior. The hippocampal theta rhythm is the prominent periodic rhythm observed in behaviorally active states and rapid-eye movement sleep. Walking is the most well-known behavior that is accompanied by prominent theta oscillation [20], [21]. Theta is also observed during immobile states if the animal is in an aroused or attentive state such as during conditioning [22], [23]. However, under behaviorally inactive states (with neither arousal nor an attentive state) such as immobility, grooming, eating, and slow-wave sleep, large irregular amplitude activity (LIA) predominates instead of the theta rhythm [20]. The state transition between these two rhythms can be detected easily by recording the field potential. Berry and colleagues have shown that the learning speed of standard eyeblink conditioning is predicted by the level of predominance of the theta rhythm in the rabbit hippocampal LFP recorded before the conditioning session [24], [25]. Furthermore, they showed that administering each trial contingent on theta expression accelerates the learning in standard eyeblink conditioning [26]. These studies clearly show the strong relationship between the hippocampal theta and the learning rate during eyeblink conditioning. However, it is not clear so far whether a correlation between the hippocampal state and the expression of CR in eyeblink conditioning exists on a trial-by-trial basis. In a conditional discrimination task in eyeblink conditioning [16]–[19], discriminative behavior in a response to an identical tone occur within one session in an individual subject. Thus, it is highly probable that top-down modulation takes place on a trial-by-trial basis. Though there have been many former studies reporting the involvement of the hippocampus in occasion setting [27]–[30], few studies have examined the physiological activity of the hippocampus during a conditional discrimination task (occasion setting) in eyeblink conditioning. The relationship between the ongoing hippocampal state and the top-down modulation of discrimination on a trial-by-trial basis in motor learning remains to be elucidated.

In the present study, we describe a strong trial-by-trial correlation between the conditioned eyeblink reflex and the hippocampal state during a conditional discrimination task. We recorded the local field potential (LFP) in the hippocampus during a serial feature positive conditional discrimination task in eyeblink conditioning in order to elucidate the neurodynamics underlying the top-down modulation. In this task, a tone (CS) paired with periorbital electrical stimulus (US) was presented when there was a preceding light stimulus, but an identical tone stimulus (CS) was presented without periorbital electrical stimulus (US) when the preceding light stimulus was not presented. This task is very similar to that used in the previous human studies [17]–[19], but we utilized a longer interstimulus interval averaging 4 seconds between the end of the conditional cue and CS onset to record ongoing hippocampal activity during the stimulus-free period. This task design with a long intervening stimulus-free period made it plausible that the task-related pre-CS activity was not evoked directly by ongoing conditional stimulus presentation, but rather reflected a change in the internal state of neural activity. We found that the animals exhibited different responses according to whether or not the preceding light stimulus had been presented. Further, the expression of the CR was dependent on the hippocampal theta elicited by the preceding light stimulus.

## Materials and Methods

The subjects used in this study were 5, 10-week-old Wistar/ST male rats (Japan SLC, Inc., Hamamatsu, Shizuoka, Japan). The rats were implanted with electrodes. After recovery, they were trained in a conditional discrimination task in eyeblink conditioning. All subjects were housed individually in standard plastic cages with free access to food and water in a colony room with a 12-h light/dark cycle.

### Ethics statement

All of the experimental procedures were performed in accordance with the NIH Guide for the Care and Use of Laboratory Animals and approved by the Experimental Animal Committee of the University of Toyama (Authorization No. 2007G-4). Throughout the experiments, all efforts were made to minimize suffering.

### Surgical procedures

A pair of twisted Teflon-coated stainless steel wires (140 µm in diameter, No. 7910, A-M Systems, Carlsborg, WA, USA) was chronically implanted in the right dorsal hippocampus under anesthesia with sodium pentobarbital (65 mg/kg i.p., Kyoritsu Seiyaku, Tokyo, Japan). Isofluran (1–2%, Abbot Japan, Osaka, Japan) was also used when necessary. The coordinates of the implants were 3.8 mm posterior to bregma, 2.5 mm lateral from the midline, and 2.5 mm dorsoventral from bregma [31]. The final depth of the electrode was determined based on the LFP recorded during the implantation. A ground electrode was connected to the stainless steel screws attached on the skull. The animals were then injected with ampicillin (100 mg/kg i.p., Meiji Seika, Tokyo, Japan) and warmed until they moved spontaneously.

After 2 weeks of recovery, surgery was again performed on each rat to implant four Teflon-coated stainless-steel wires (140 µm in diameter, No. 7910, A-M Systems, Carlsborg, WA, USA) in the left upper eyelid to record electromyographic (EMG) activity and to deliver a periorbital shock as the US. These wires were soldered to connector pins, which were secured to the skull with dental acrylic resin and stainless steel screws.

### Stimuli and procedure

Two to four days after the eyelid surgery, the animals were given 2 days to adapt to the experimental apparatus. These adaptation sessions were performed in the same way as the conditioning sessions except that no stimuli were presented. EMG and LFP data were recorded to calculate spontaneous eyeblink frequency and spontaneous hippocampal theta activity, respectively. The timings of stimuli were generated in the same way as in the conditioning sessions, but without actual deliveries, to calculate spontaneous EMG activity and hippocampal activity.

**Figure 1:**
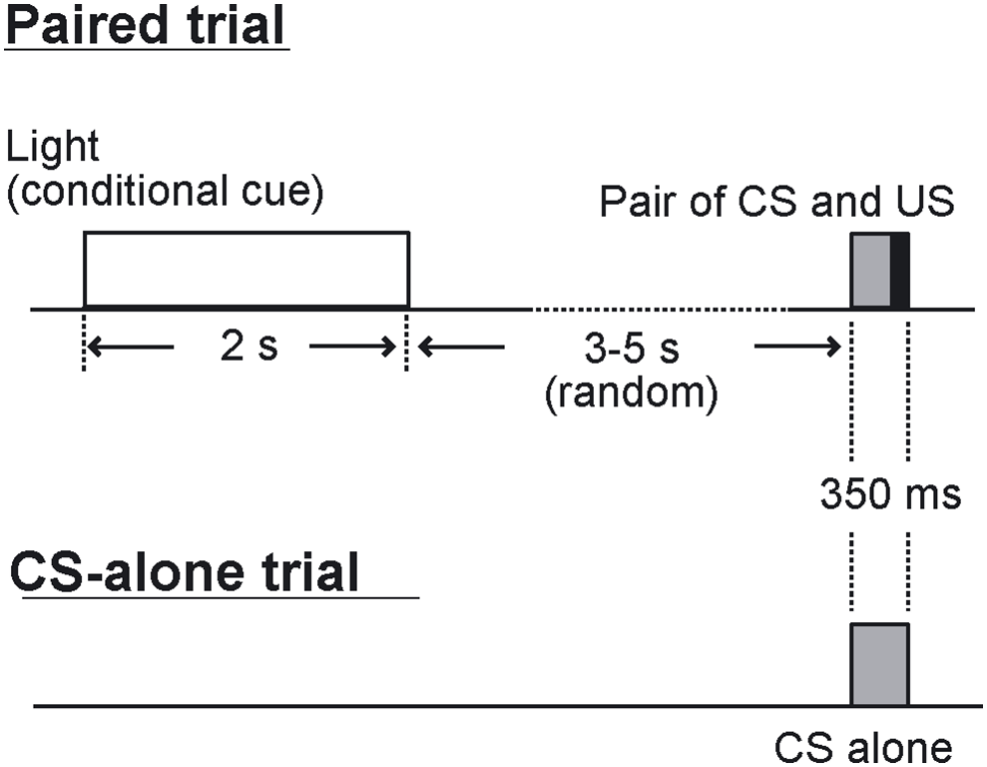
The sequence of the stimuli in the conditional discrimination task. Two kinds of trials were used in the task: paired trials, and CS-alone trials. In a paired trial, a chamber light stimulus is delivered before the CS. The duration of the interval between the end of the light stimulus and the CS onset is 3 s to 5 s, averaging 4 s. In a CS-alone trial, the CS alone is delivered without reinforcement.

After the adaptation sessions, the conditioning sessions were performed for 10 days. We used two types of trials: paired trials, and CS-alone trials (Fig. 1). In both types of trials, an identical tone (350-ms, 1 kHz, 85–90 dB) with a rise/fall time of 10 ms was used as the CS. In a paired trial, a light stimulus (533 cd) with duration of 2 s was delivered 5–7 s before the CS as a conditional cue, resulting in a 3- to 5-s, stimulus-free interval between the end of the conditional cue and the onset of the CS. This variable and relatively long stimulus-free interval between the light cue and the CS was adopted to prevent the rats from acquiring a conditioned response to the light stimulus, because a stimulus-free interstimulus interval longer than 1 s is difficult to learn in eyeblink conditioning [32]. The CS was paired with a 100-ms periorbital shock US (1.5 mA, 100-Hz square pulses) that was delivered through a pair of electrodes implanted in the left upper eyelid. The interstimulus interval between the CS and US was 250 ms. In a CS-alone trial, the CS was delivered without the conditional cue and the US.

A rat was placed in a cylindrical Plexiglas container with a 16-cm diameter and 35-cm height set in a sound- and light-attenuated chamber. A light source made of 70 light-emitting diodes was positioned on the ceiling of the chamber. A daily session consisted of 100 trials, which included 50 paired trials and 50 CS-alone trials. Each session was divided into 50 successive groups of two trials, each of which consisted of a paired trial and a CS-alone trial. The order of the trials in each group was random. Trials were separated by a variable intertrial interval randomized over 30–40 s with a mean of 35 s.

### Electrophysiological recordings and data analysis

Electrophysiological measures were recorded every session throughout the behavioral training. The hippocampal LFP and eyelid EMG were amplified (×2000) and recorded at a sampling rate of 7,575 Hz with a Neuralynx Cheetah 32-channel system (Neuralynx, Tucson, AZ, USA). During this process, the stimulus event markers were recorded simultaneously. The analog filter was set at 1–475 Hz for the LFP signal and 100–3000 Hz for the EMG signal.

To assess the discriminative learning performance of rats, we separately calculated the frequency of occurrence of CRs in each trial type (CR percentage). The EMG signal was processed as described below to calculate the CR percentage. Firstly, the raw signals were converted to new signals called EMG amplitude signals. The value for EMG amplitude at a given time was calculated from the raw signal by assigning to every time point *t* the absolute difference between the maximum value and minimum value within the ±1-ms time window surrounding this time point. This process filtered away low frequency components and generated non-zero signals, which are more easily handled. Then, the sum of the mean value and the SD of the EMG amplitude for the pre-CS periods (0–300 ms before CS onset) in 50 trials of the same trial type were defined as the threshold, which was then used in the analysis below. Next, invalid hyperactive trials were defined. For this, the threshold value was subtracted from the EMG amplitude and the negative values were assigned as zero again. Then, the average value of this time series was calculated for the pre-CS period for each trial. If this value exceeded 10% of the threshold value, the trial was assigned as an invalid trial and excluded from the calculation of the CR percentage. A trial was assigned as having an adaptive CR if the average value of the subtracted value for the 200-ms period before US onset exceeded 10% of the threshold and exceeded 10 times that of the pre-CS period. The percentage of trials with adaptive CRs within all valid trials in a session was calculated and denoted as the CR percentage. The CR percentage difference between the paired and the CS-alone trials was calculated by subtracting the CR percentage calculated for the CS-alone trials from that for the paired trials. To compare the learning performance between trial types, we used analysis of variance (ANOVA) with repeated measures.

The normalized CR amplitude signal for each trial was calculated by normalizing the average EMG amplitude across a 200-ms interval before the US onset by the spontaneous EMG amplitude of the session. The spontaneous EMG amplitude was defined as the mean EMG value for the pre-conditional-cue interval (0–300 ms before cue onset).

### LFP analysis

First, the sampling rate of all LFP data was reduced from 7,575 Hz to 1,515 Hz off-line using the CSC File Rate Reducer program (Neuralynx, Tucson, AZ) to reduce unnecessary computational cost. The signals were inspected to discard trials containing movement artifacts (<4% of trials), which are easily recognizable by a simple visual examination of raw signals. Spectral analyses were carried out using the multitaper FFT MATLAB package by Mitra and Pesaran [33]. The power spectra were calculated over frequencies ranging 1–30 Hz. For dynamic spectral analysis, the FFT window length was 2 s with a stepping width of 0.1 s. For calculating the relative theta power, the sum of the power for theta band frequency (5–10 Hz) was divided by the sum of the power over frequencies 1–30 Hz. In the paired trials, the shortest interval between the end of the light cue and onset of the CS was 3 s. Thus, we used the FFT window length of 3 s (Fig. 1).

We compared the relative theta power during the pre- and post-stimulus 3-s intervals between the different trial types. The relative power of the theta band depends largely on whether the hippocampus is in the state of theta rhythm or LIA. The 3-s intervals in each trial type contained both theta dominant trials and LIA dominant trials.

The correlation coefficient between the CR amplitude and relative theta power was calculated for each session in each animal using all trials. To evaluate the correlation between theta power and trial type, the correlation ratio (equivalent of the correlation coefficient for categorical data) was calculated for each session and each animal by dividing the between-class variation by total variation of relative theta power.

### Histology

The loci of electrode tips were examined after completion of conditioning sessions. The animals were anesthetized with sodium pentobarbital (65 mg/kg i.p., Kyoritsu Seiyaku, Tokyo, Japan), and electrical current (−50 µA, 30 s) was passed through the tips of the electrodes. After 2–3 days, rats were deeply anesthetized with a lethal dose of pentobarbital and perfused transcardially with 0.9% saline, followed by 10% formalin. The brains were removed and placed in 10% formalin. Prior to sectioning, they were placed in a 30% sucrose solution overnight. Frozen sections (40-µm thick) were prepared, and the loci of the tips of the electrodes were examined under a microscope.

## Results

### Histology

**Figure 2:**
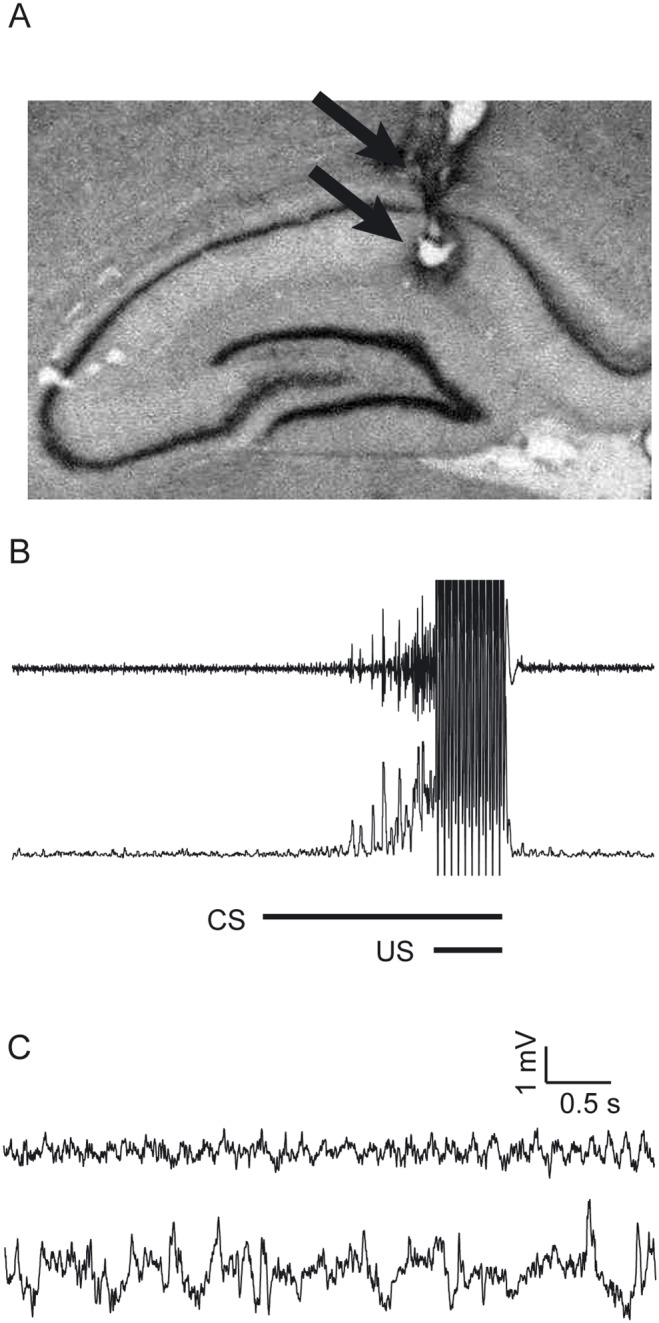
Electrode position in hippocampus. (A) The picture of a brain section taken from one of the animals. The arrows show the tips of a pair of the electrodes. (B) The upper trace shows the raw EMG signal around the CS of a paired trial. The lower trace shows the EMG amplitude signal calculated using the method described in the text. (C) Representative raw hippocampal LFP signals recorded by a pair of electrodes in a same session showing theta oscillation and large irregular amplitude activity (LIA). The upper trace shows representative theta oscillation and the lower trace shows a representative LIA.

All 5 subjects had recording electrodes successfully placed in the dorsal hippocampus. Figure 2A shows a representative location of the tips of a pair of the electrodes in the hippocampal slice.

### Behavioral result

**Figure 3:**
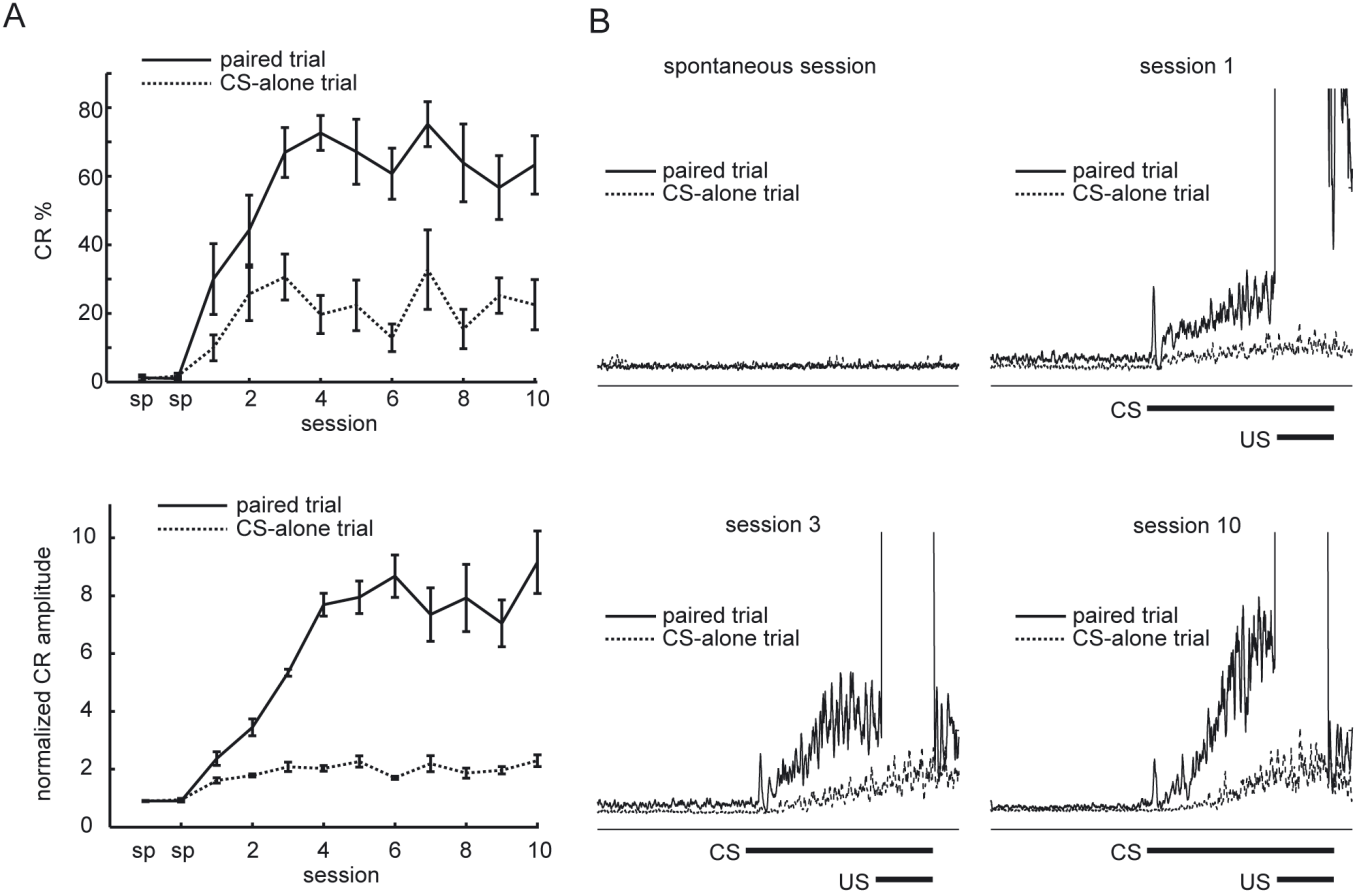
Acquisition of discriminative CRs. (A) The upper figure illustrates the averaged learning curves for 5 rats calculated for each type of trials. Vertical bars indicate the standard error of the mean. The lower figure shows the learning curves illustrated by the average normalized CR amplitude across all rats calculated separately for each type of trials and session. Vertical bars indicate the standard error of the mean. (B) Averaged normalized EMG amplitudes for paired trials and CS-alone trials of a rat for different sessions. sp, spontaneous session.

The conditional discrimination task in eyeblink conditioning was performed over two adaptation sessions and 10 consecutive acquisition sessions. The learned responses could be clearly detected by the EMG recording of the eyelid (Fig. 2B). Figure 3A shows the averaged learning curves for both trial types. The rats showed progressively increasing CR percentage values both in paired trials and CS-alone trials. However, the rats showed higher CR percentage in the paired trials.

The average CR percentage value of the paired trials increased up to 62.4±7.7%, whereas the average CR percentage value for the CS-alone trials increased up to 25.7±10.0%. Thus, the rats acquired differential CRs to the identical tone CS according to the presence of the preceding conditional light stimulus. A comparison across the 10 sessions using a two-way analysis of variance (ANOVA) with repeated measures revealed an effect of sessions, (*F*(9, 80) = 2.75, *P* = 0.0074), and trial type, (*F*(1, 80) = 110.0 *P*<0.001), but no interaction between sessions and trial type, (*F*(9, 80) = 1.40, *P* = 0.20). The lower panel of Figure 3A shows the average of normalized CR amplitudes calculated separately for both trial types. A comparison across the 10 sessions using a two-way ANOVA with repeated measures revealed an effect of sessions, (*F*(9, 80) = 2.11, *P* = 0.038), and trial type, (*F*(1, 80) = 81.6 *P*<0.001), but no interaction between sessions and trial type, (*F*(9, 80) = 1.66, *P* = 0.11).

Figure 3B shows EMG amplitude averaged for each trial type acquired from the spontaneous session, session 1, session 3 and session 10 of a rat (rat t2).

### Hippocampal local field potentials

Hippocampal field signals were visually inspected trial-by-trial to discard data containing artifacts by strong physical jerks of the animal (<4% of trials). The LFP data of session 4 of two rats (rat “t5” and “t6”) were discarded from the analysis because of a recording problem from the LFP electrodes. Prominent hippocampal theta oscillations were observed in the LFP signals. Theta oscillations and non-theta irregular activity could easily be distinguished by observation of the LFP signal (Fig. 2C).

### Hippocampal theta is elicited by presentation of conditional cue

**Figure 4:**
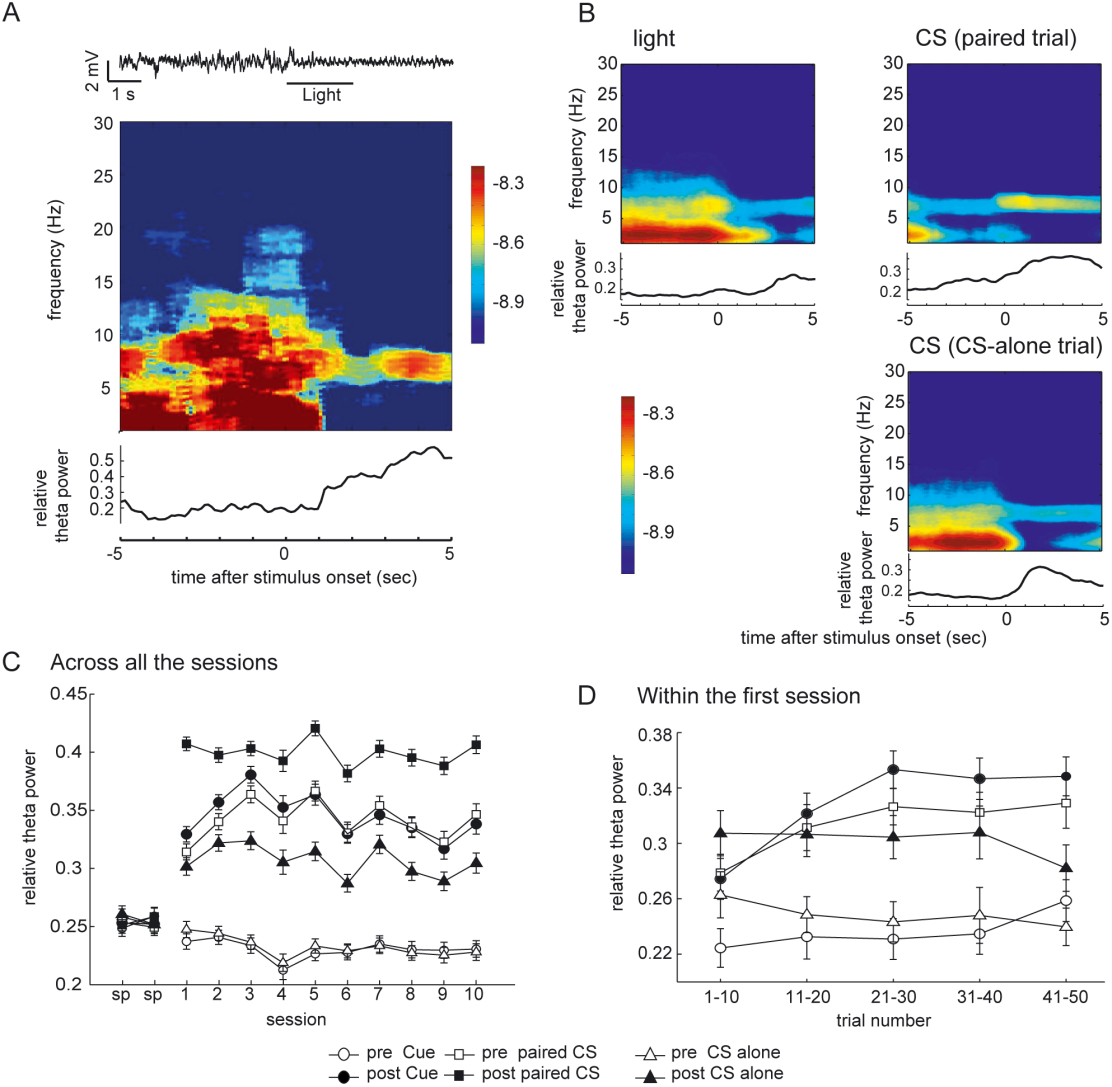
Elicitation of the hippocampal theta by the stimuli. (A) (B) The dynamic power spectra showing the elicitation of theta oscillation by the stimuli. Note that the main reason for the predominance of the theta rhythm is the decreased power of frequency ranges other than theta, while the absolute power of the theta range remained constant. (A) A representative LFP signal around a presentation of the light cue. The theta oscillation is elicited by the presentation of the cue. The middle panel shows the dynamic power spectra for the same trial using a 2-s moving window. The lowermost panel trace shows the relative power of the theta frequency range calculated for each time point using the moving window centered at that time. (B) Dynamic power spectra around the stimuli and the relative theta power. Theta band activity is evident after presentation of both the conditional light cue and CS. Shown are the averages across all the trials for each stimulus type in the session 10 of a rat (50 trials each). x axis, time (in s); y axis, frequency (in Hz). The color scale represents the signal power (in log_10_V^2^). The width of the moving time window is 2 s long. The relative theta power was calculated with the average dynamic power spectra showed above the trace with 2-s time window. (C) The relative theta power (5–8 Hz) calculated for each 3-s prestimulus and poststimulus interval. The average relative theta power over all valid trials from the same day across 5 rats. Vertical bars indicate the standard error of the mean. (D) Same data from the first acquisition session calculated for successive blocks of 5 trials. The data for post paired CS period are excluded. Vertical bars indicate the standard error of the mean.

Stimuli typically elicited theta rhythms in the hippocampus. Figure 4A shows a representative transition of the state of the hippocampal LFP from irregular activity to theta rhythm triggered by the conditional light cue. As can be seen in the dynamic spectra from the same trial, these distinctive states are apparent in the difference between the power spectra. It is not the case that the absolute power of the theta frequency range is larger in the theta state versus the non-theta state (LIA). Rather, the power in the non-theta frequency ranges decreases when the theta rhythm predominates, leading to periodic activity with higher relative theta power. To assess changes in spectral properties following the presentation of stimuli, we calculated the dynamic power spectra around stimulus onset of the conditional cue, CS of in the paired trial, and CS in the CS-alone trial. We found that conditional light stimuli, the pair of CS and US, and CS-alone all evoked hippocampal theta rhythms (Fig. 4B).

Figure 4C illustrates the average relative theta power across all 5 rats for each session, stimulus, and trial type for both pre- and post-stimulus intervals. The prestimulus interval and poststimulus interval were defined as the 3-s interval before the onset of the stimulus and the 3-s interval after the end of the stimulus, respectively. The values for the post-paired CS interval were reliably and stably high across all sessions. The ability of the conditional cue to elicit theta rhythm was already evident in the first day of conditioning (session 1) and reached the asymptotic level by session 3, as is shown in the post-cue value. The value for the pre-paired CS intervals that followed a few seconds later, was slightly less than that of post-cue interval in the first half of the experiment (sessions 1–5), but slightly higher in the latter half of the experiment (sessions 6–10). The values of pre-cue and pre-CS-alone trials stayed at baseline levels across all the conditioning sessions, with slightly less power than in the adaptation sessions. A comparison across the 12 sessions using a two-way analysis of variance (ANOVA) revealed an effect of sessions (*F*(11, 331) = 7.24, *P*<0.001), and interval type (*F*(5, 331) = 79.86 *P*<0.001).

To investigate early changes in the hippocampal LFP, data from the first session for each trial type other than post-paired trial were subdivided into 10 consecutive blocks, each of which comprised 5 consecutive trials (Fig. 4D). The elicitation of theta rhythm by the light stimulus increased over the first 30 trials and then became saturated. The relative theta power of the pre-CS interval in paired trials paralleled the data of the post-cue period. A comparison across the 50 trials using a two-way analysis of variance (ANOVA) with repeated measures revealed an effect of trials (*F*(1200, 49) = 1.77, *P* = 0.001) and interval type (*F*(1200, 5) = 55.18 *P*<0.001), but no interaction between sessions and trial type (*F*(1200, 245) = 0.72, *P* = 0.99).

### Correlation between CR expression and pre-CS hippocampal theta state

**Figure 5:**
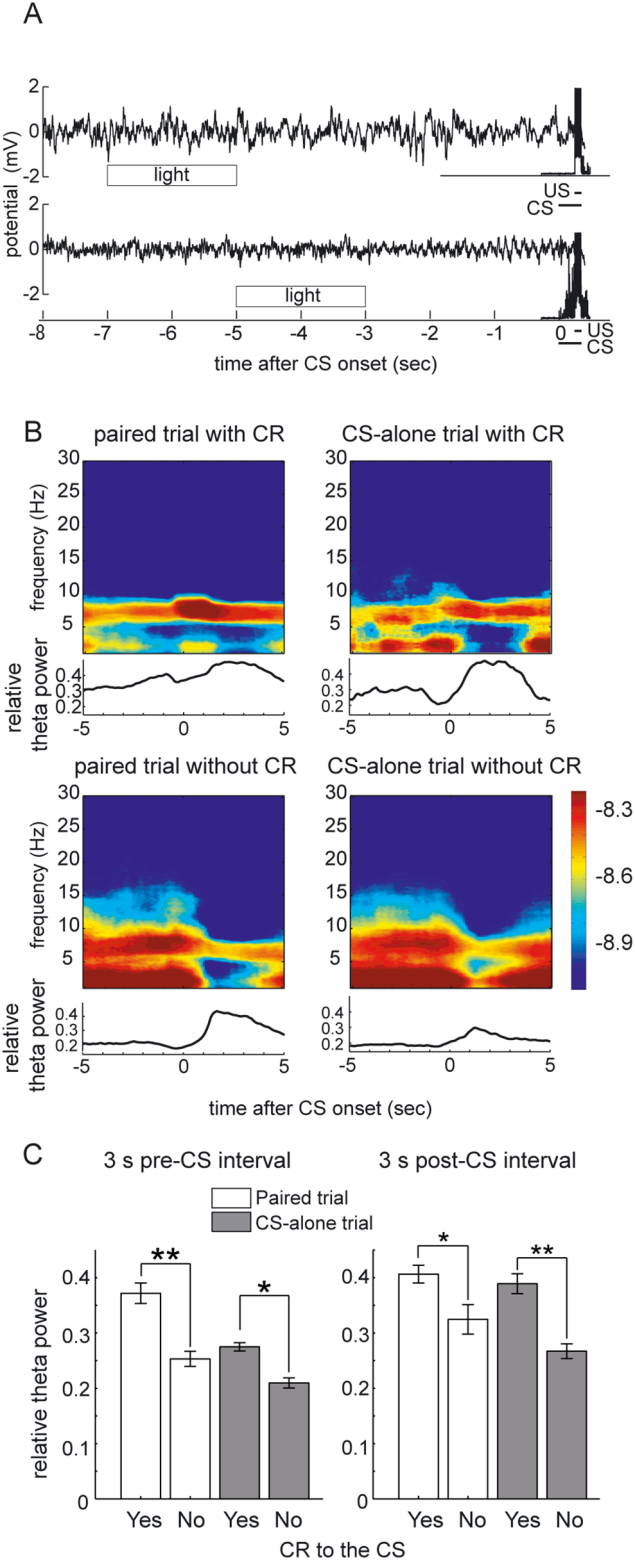
Dependency of the CR expression on the state of hippocampal LFP before the CS onset. (A) LFP signals and EMG amplitude signals for two successive paired trials (trial 24 and trial 25 in session 10). The theta rhythm is absent and no CR response is observed in the former trial. A prominent theta rhythm and successive CR response are observed in the latter trial. (B) Averaged dynamic power spectra around the CS presentation calculated separately according to the trial type and presence of CR. Theta band activity is evident before the presentation of CS in the trials with CR expression. Shown are the data from the session 10 of a rat. x axis, time (in s); y axis, frequency (in Hz). The color scale represents the signal power (in log_10_V^2^). The width of the moving time window is 2 s long. The lower traces corresponds the relative theta power calculated from the average dynamics spectra shown above. (C) The average relative theta power across the last 5 sessions (sessions 6–10). All the valid trials in those sessions for each rat were separated according to the trial type and presence of the CR to the CS for the calculation of the relative theta power. Then, the average relative theta power across all the rats was compared. Significant difference were observed between CR trials and no-CR trials (paired *t*-test, **P<0.001; *P<0.005). Vertical bars indicate the standard deviations.

Theta oscillations were not always elicited by the conditional cue in the paired trials, even after learning had reached the asymptotic level, and rats sometimes wrongly expressed CRs in the CS-alone trials. Because of this variability, we further examined whether the hippocampal state at the time of CS delivery affected the subsequent response to that CS on a trial-by-trial basis. Figure 5A shows two sets of data in the successive paired trials of an animal (trial 24 and trial 25 in session 10), which showed a different hippocampal state at the time of CS delivery as revealed by the presence or absence of the theta rhythm in the hippocampal LFP signals. In trial 24 (upper traces), the animal showed neither the hippocampal theta rhythm before the CS nor any responses to the CS. On the contrary, in trial 25, prominent theta rhythm elicited by the preceding light stimulus was evident before the presentation of the CS. On this trial, the stimuli evoked a large CR. To confirm the relationship between the hippocampal state prior to CS presentation and its subsequent CR expression, dynamic power spectra of the hippocampal LFP around the CS onset were calculated in the same way as in Figure 4B, except that the paired trials and the CS-alone trials were further subdivided into those with or without the CR (Fig. 5B). We found that, in addition to the paired trial with correct CRs, the CS-alone trial with incorrect CRs also showed a theta range spectral peak before the CS, which was further strengthened after the CS (upper panels in Fig. 5B). In contrast, the CS-alone trials that correctly showed no CRs, as well as the paired trials without the CRs by mistake, exhibited much broader spectral peaks for the hippocampal LFP. These LFPs corresponded to large irregular amplitude activity (LIA) before the CS (lower panels in Fig. 5B). This broader spectrum then converged on the theta range for a while after the CS.

We conducted statistical comparisons for the relative theta power in the 3-s pre-CS interval between trials with adaptive CR responses and trials without adaptive CR responses. In order to analyze the behavioral asymptotic phase, the data of the latter half of the sessions (sessions 6–10) were used. Invalid trials defined by the electromyographic hyperactivity or artifacts in LFP signals as described above were discarded. All of the valid trials from sessions 6–10 of each rat were divided according to the presence of CR and trial type. Then, the relative theta power for each pre-CS interval was calculated for each trial, and compared (Fig. 5C). There were significant differences between the relative theta power of the pre-CS intervals between trials with CRs and trials without CR, both for paired trials and CS-alone trials (paired *t*-test, paired trials: *P*<0.001; CS-alone trials: *P*<0.005). Because we observed a difference in the elicitation of theta rhythm by the CS stimulus according to the presence of the CR, we did the same analysis for the post-CS intervals as well (Fig. 5C). There were also significant differences between the relative theta power of the post-CS intervals between trials with CRs and trials without a CR, both for paired trials and CS-alone trials (paired *t*-test, paired trials: *P*<0.005; CS-alone trials: *P*<0.001).

### Correlation between the relative hippocampal theta power and the CR amplitude paralleled with acquisition of discriminative CR

**Figure 6:**
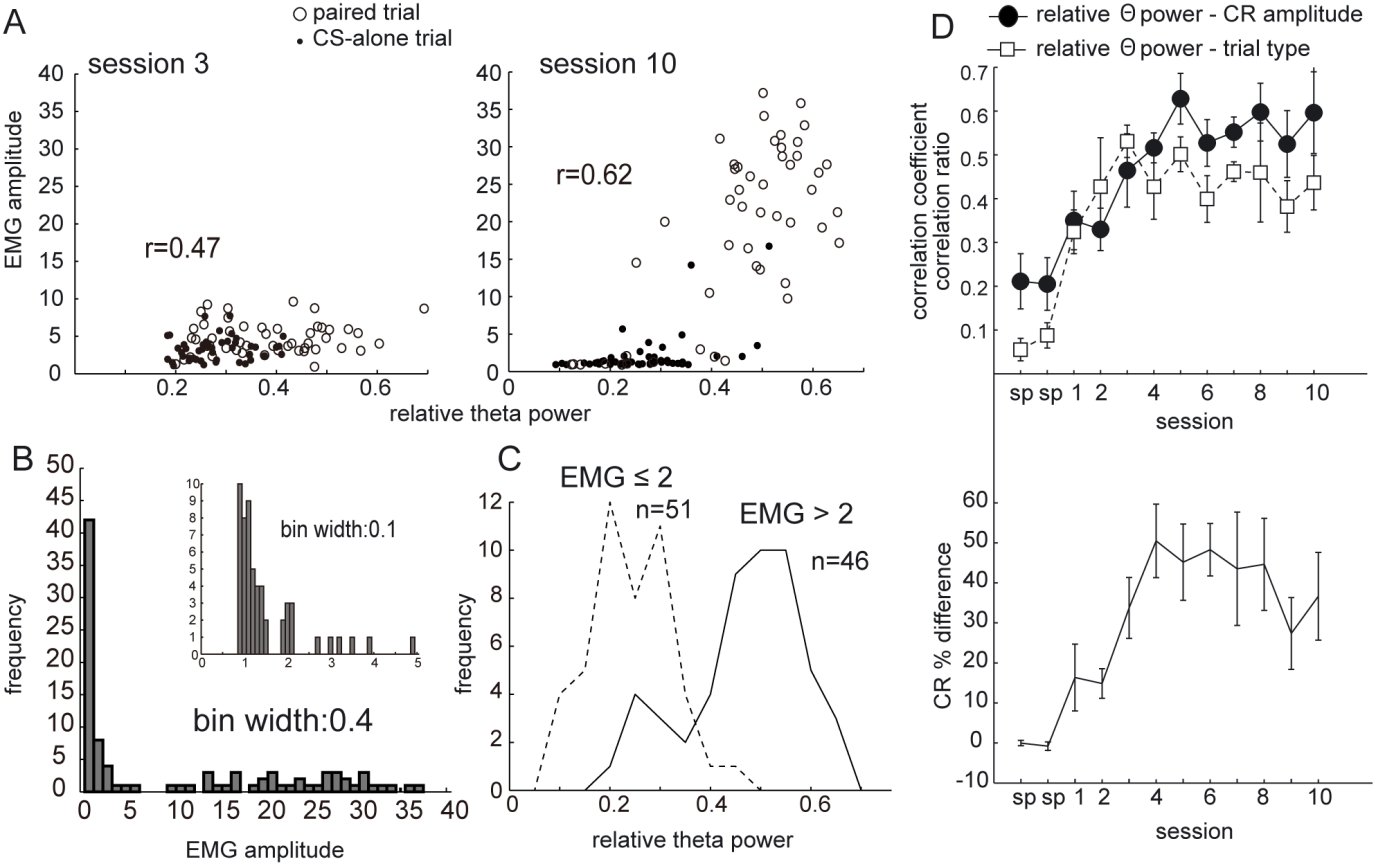
Correlation between CR amplitude and theta power. (A) Scattergrams showing the relationship between CR amplitude and relative theta power. Each point indicates each trial. Open circles correspond to paired trials and filled circles correspond to CS-alone trials. Shown are data from one rat (rat t6). x axis, relative theta power of 3-s prestimulus interval calculated for each trial; y axis, normalized CR amplitude. (B) Histogram of CR amplitude for session 10 from the same rat. Sharp peak around the value 1 corresponds to the trials without any response to the CS showing spontaneous level of EMG. The distribution shows a clear bimodal clustering tendency. (C) Histogram of relative theta power of prestimulus intervals for session 10. The trials were divided according to whether the EMG amplitude is bigger or smaller than 2. (D) The upper panel shows the plots of average correlation coefficients between CR amplitude and relative theta power for the 5 rats, and average correlation ratio between trial type (paired trial or CS-alone trial) and relative theta power for prestimulus interval for the 5 rats. The lower panel shows the plot of average CR percentage difference between paired trial and CS-alone trial for 5 rats. Each value was calculated for each session, and then the average and standard error were calculated across 5 rats.

To quantitatively evaluate the relationship between the hippocampal state and CR expression, we examined the correlation between the relative theta power before the CS and the subsequent amplitude of the EMG integrated over a 200-ms interval before the US (Fig. 6). Figure 6A compares the relationship between the relative theta power and the EMG amplitude within a session between behaviorally early phase (session 3) and later phase (session 10). The correlation coefficient between the relative theta power and EMG amplitude was 0.47 in session 3 and smaller than 0.62 in session 10. The scattergrams illustrate that the EMG amplitude distribution was positively skewed. The histogram of the normalized EMG amplitudes at the asymptotic phase showed that while a large portion of the trials had amplitudes around the value 1.0, the values for other trials were distributed rather broadly, as shown in Figure 6B. This corresponds to the fact that when the animals did not show any response to the CS, the EMG amplitude was equivalent to the spontaneous value 1.0, while the CR amplitude had higher variability. Figure 6C shows the differences in the histograms of the relative theta power for the pre-stimulus interval of the CS based on whether the EMG amplitude was bigger or smaller than the value 2. When the EMG amplitude was smaller than 2, most of the trials had the relative theta power smaller than 0.3 (218/274 = 79.6%, session 10 across all the rats).

To investigate the development of discriminative behaviors, the differential expression of theta rhythm just before the CS, and the dependency of CR on the LFP, we plotted the CR percentage difference between the two trial types, the correlation ratio between trial type (paired trial or CS-alone trial) and relative theta power, and the correlation coefficient between CR amplitude and relative theta power (Fig. 6D). The correlation coefficient between the CR amplitude and relative theta power was calculated for each session of each animal using data from both types of trials. Then, these coefficients were averaged for all 5 rats. The correlation ratio (equivalent of the correlation coefficient for categorical data) was used to evaluate the correlation between theta power and trial type. The correlation ratios were calculated for each session of each animal as the ratio between the sum of squares between trial types and the total sum of squares, and then averaged over all 5 rats. The difference in CR percentage was calculated for each session for each animal by subtracting the calculated CR percentage of the CS-alone trials from CR percentage of the paired trials. These data were then averaged across 5 rats for the same session number. The correlation ratio between the trial type and relative theta power progressed to the asymptotic level within the first 3 sessions (Fig. 6D). However, the behavioral discrimination and the dependency of the CR expression on the LFP state developed over 4–5 sessions (Fig. 6D).

## Discussion

In the present study, we investigated the change in hippocampal local field potentials during a serial feature positive conditional discrimination task in eyeblink conditioning in order to elucidate the neurodynamics underlying the top-down modulation observed in eyeblink conditioning. We observed that rats had the ability to discriminate by showing conditionally flexible conditioned responses, that hippocampal theta was elicited by the conditional light cue, and that the expression of a CR correlated with whether the preceding state of the hippocampus was exhibiting theta rhythm when the CS was presented. Our results suggested that that the hippocampus is functionally involved in the realization of top-down modulation of the elicitation of the CR and that the hippocampus is possibly modulating the cerebellum and its associated circuitry during such tasks.

### Discriminative CR expression following the same tone stimulus

Behavioral results show that animals can acquire differential conditioned responses to the same tone stimulus according to the presence of a preceding light stimulus. Some previous studies with human subjects have utilized a similar conditional discrimination task for elucidating the effect of temporal lobe lesions [17], [18], age [34], or stress exposure [19]. The present study demonstrates that further research utilizing conditional discrimination tasks in eyeblink conditioning can be conducted using an animal model. This allows for several experimental advantages, such as the possibility of using electrode implantation, pharmacological reagents, or systematic lesions. Another trial-by-trial contextual discrimination task using eyeblink conditioning showed that rabbits showed differential responses to the same tone according to the ambient light in the chamber [15]. In addition, strong top-down modulation of the expression of acquired standard conditioning in standard delay paradigm has also been reported [14]. However, the present experimental setting differed in that no contextual stimulus was being presented immediately before the onset of the CS. In our study, we showed that rats were able to acquire this task in spite of the long interstimulus interval between the end of the conditional cue and the onset of the CS. The characteristics of the task design would make it easier to separate the neural activity evoked directly by the conditional cue stimulus and the change in internal neural dynamics, which may last several seconds after termination of the cue stimulus. Information regarding context should thus be stored in the brain in the intervening delay period between the end of the conditional light cue and the CS onset in order to discriminate between the consequences of the CS. In other words, the reason for differential responses to the same tone is attributable to differences in internal brain state at the time of tone presentation. Thus, this process requires a mechanism (or neural circuitry) for retaining the conditional information during the temporal gap. The transition of the hippocampal state to the theta oscillation observed here possibly reflects the relevant internal state that provides conditional information and the ability to modulate sensorimotor integration.

### Elicitation of hippocampal theta by conditional cue

In our present study, the rat typically showed attentive behavior on the presentation of conditional stimuli, by moving the body slightly, gazing upright, and moving the neck slowly in the opposite direction to the implanted eye. However, the rats were immobile most of the time during the inter-stimulus interval. This is consistent with the fact that the spectral peak of the observed hippocampal theta is around 5–8 Hz, which is the characteristic frequency band for “type 2 theta” [22], [23]. Type 2 or “atropine-sensitive theta” is generally associated with an immobile, attentive state.

As illustrated in Figure 4C, the elicitation of hippocampal theta by the light and tone stimulus was already evident within the first session. This can be interpreted as fast acquired salience of these neutral stimuli by the presentation of paired trials. Moreover, the relative saliency in terms of theta elicitation for the tone stimulus and light stimulus alternate in the first half of the first session. Namely, the elicitation of the theta by the CS in CS-alone trials is larger than that of the light stimulus in the very first 10 trials, but this pattern is reversed in latter trials. This may reflect that the saliency of the stimuli shifts from pure association of tone and US to the predictive value of the light stimulus. The largest elicitation of theta in the poststimulus interval of the paired CS over all the sessions could be interpreted as the consequence of noxious US delivery [20].

### Possible functional role of the hippocampus in conditional eyeblink conditioning

The hippocampus is essential for the disambiguation of sequences that share common events [35]–[37]. It is also involved in bridging temporal gaps [35], [38], [39]. Consistent with these arguments, some studies have suggested the involvement of the hippocampus in conditional discrimination tasks in eyeblink conditioning [17]–[19], as well as in occasion setting [27]–[30]. In accordance with these observations, we found a strong correlation between the hippocampal state and behavior in the present study. Specifically, the elicitation of theta rhythm and the trial-to-trial correlation between the appearance of the hippocampal theta and the expression of the CR in the behaviorally asymptotic phase were observed. As far as we know, this kind of trial-by-trial correlation between the theta oscillation and the expression of reflex has not yet been reported.

A vast amount of previous research has identified the basic circuitry for eyeblink conditioning in the cerebellum and its interconnected brainstem nuclei [7], [9], [10]. In addition, some types of eyeblink conditioning paradigms involve the circuitry in the forebrain such as prefrontal cortex [13], [40], hippocampus [5], [12], [26], [40], [41], and amygdala [42]–[44]. Along with these studies, our current study suggests that the dynamics of the cerebellum mediating the eyeblink reflex may be modulated by the ongoing hippocampal state. The possible dependency of the dynamics of the cerebellum and its associated circuitry on the hippocampal state suggests that the coordinated state transition of the brain between the mode for communicating with external environment and the mode for internal processing includes cerebellar function. The hippocampus is thought to have at least two distinctive states: the theta state with ongoing periodic LFP oscillation and the non-theta state with intermittent bursts of sharp wave–ripple complexes embedded in LIA. It is well known that the transition between these states corresponds to the ongoing behavior. Hippocampal theta rhythm reflects the “on-line” state of the hippocampus, related to the active behavior such as walking, anticipation, arousal, and conditioning and subjective states described in terms such as “voluntary,” “preparatory,” “orienting,” or “exploratory” [23], [45]. On the other hand, LIA is observed while the animal is in behaviorally quiescent states such as grooming, eating, and sleeping [20]. The physiological properties of hippocampal neurons substantially differ between these two states. During the theta rhythm, principal cells in the hippocampus show sparse representations of the environment as observed in place cells [46], while the same cells can show intermittent and synchronous bursts of activity with concurrent LFP activities known as the sharp wave–ripple complex [47], [48]. There have been numerous theories about the behavioral correlates of the theta rhythm, such as orienting [49], voluntary behavior [45], and sensorimotor function [50]. Most of these behavioral correlates fall into either of two general categories: attention to sensory input, or motor output [21]. In these theories, the theta rhythm appears when the brain is interacting with the surrounding environment. On the other hand, there is a growing consensus that the functional role of the non-theta state is associated with systems consolidation processes [51]. Assuming that systems consolidation relies on communication between the neocortex in slow-wave sleep and the hippocampus in its non-theta state, the non-theta state is characterized by communication taking place between multiple systems within the brain, rather than interacting with the external environment. In accordance with this view, the conditioned response, which includes both the processing of sensory information and motor output, appeared in the presence of the theta rhythm in the present study, irrespective of the preceding contextual cue.

In our present study, the change in the hippocampal state was detected by the change in the power spectrum of the hippocampal LFP. This change was characterized by a large decrease in the power of the frequencies out of theta range, whereas the absolute power of the theta frequency range itself did not show large differences (Fig. 4A). However, taking into consideration that the hippocampus is likely to function differently in a different mode of oscillation, we interpret the current result as a correlation between CR expression and the hippocampal state (dominance of theta oscillation), rather than a correlation between CR expression and decrease in power of the delta oscillation.

Although the hippocampal formation is not required for acquisition of the standard delay paradigm, there have been many studies showing the involvement of the hippocampus. Berry and colleagues showed that the learning speed is predicted by the level of theta predominance [24]. They showed that those rabbits that had shown higher relative theta power prior to the conditioning learned faster than those with lower relative theta power. Moreover, they used an experimental setting called “theta-contingent training” using a well-timed brain-machine interface to condition the animal in the theta state [26], [52]. They found that there was a large difference in the initial learning rate between the animals conditioned in the theta state and those in the non-theta state. These experiments have clearly shown the importance of the brain state, monitored by the hippocampal theta wave, on the overall performance in an early session which varies across the individuals. Some studies have shown a strong influence of context in the expression phase [5], [14]. Perturbation of the hippocampus has also been shown to have strong top-down influence on the expression of acquired CRs [5], [53]. These findings are consistent with our hypothesis that the hippocampus influences the expression of CRs. Some recent studies report phase synchrony in the theta frequency range between the hippocampus and the cerebellum [54], [55]. Nokia and colleagues reported that at behaviorally intermediate learning stages, the CR-like activity of hippocampal multi-unit activity closely models the shape of CR in trials with ongoing theta rhythm [56]. These studies support the idea that hippocampal theta rhythm is involved in the interaction between the hippocampus and the cerebellum.

In the present work, we focused on the relationship between the hippocampal state and the trial-by-trial performance, which varies across the trials within a session. We found a significant correlation between theta rhythm dominance and CR expression on a trial-by-trial basis during the conditional discrimination task. Furthermore, the correlation between CR expression and theta rhythm predominance was evident in the asymptotic level as well. These results have not been reported in former studies showing correlation between theta rhythm and behavior in a standard delay paradigm [24], [26], [52]. Nokia et al. [56] found that the CR peak latency was slightly different between the trials with high theta power and those with low theta power in the intermediate learning stage before reaching the asymptotic level of learning. However, they did not observe a difference in CR amplitude, which we found in the present study. We assume that the large correlation between the hippocampal theta and the CR expression resulted from our task design which utilized two types of conditions, forcing the animal to respond differently to an identical CS on a trial-by-trial basis, so that top-down modulation by the hippocampus might be stronger than for a standard delay paradigm, allowing appropriate output to be executed according to the context.

The correlation ratio between the trial type and relative theta power in the 3-s interval before the CS onset reached the asymptotic level within 3 sessions. On the other hand, behavioral discrimination and the correlation between the CR expression and the LFP state developed over 4–5 sessions (Fig. 6D). These results may suggest that the development of discriminative CR expression does not depend solely on the development of theta predominance in the cued paired trials, but also reflects the dependency of CR expression on the brain state, which would develop after the light stimulus acquires a behavioral saliency. Further work is needed to elucidate this relationship between the development of discriminative behavior and the development of the brain state-dependency of the expression.

The LIA typically appears during slow-wave sleep. Therefore, there is a possibility that the observed correlation between the hippocampal states and CR expressions might result from suppression of CRs by an intermittent sleep that might occur during a conditioning session. However, the non-theta state (LIA) observed in the present study does not necessarily signify slow wave sleep during a conditioning session. In fact, it is known that LIA is also observed in awake states [57], [58]. When a rat shuttles on a linear track, theta activity ceases and LIA appears during the short pause at the end of the track [59], [60]. In addition, the time for the behavioral alternation between the trials with CR and without CR seems to be too short for a transition between sleeping and awake states. The sleep onset latency of rats in the light phase of the day was reported to be about 8 min [61], while in the present study the average period of successive trials without CRs was 134 s and that with CRs was 105 s (see Figure S1 in supporting information for detail). Therefore, it is unlikely that the rats repeatedly transitioned between awake and sleeping states 50 times within a conditioning session. We also confirmed the significant correlation between the hippocampal state and CR expression after confining the data to those trials that followed immediately after the CR-expressing trials (see Figure S2 in supporting information), based on the assumption that the rats were not asleep just after they had expressed the CR.

Though the relevance of sleep to the suppression of CR should be considered, sleep is not a trivial explanation for the fluctuations in CR expression. As far as we know, there have been no studies reporting a random failure in CR expression due to an occasional sleep during an ongoing conditioning session. Furthermore, a mechanism for CR suppression by an intermittent sleep is not clear, if it exists at all. The CS is conveyed from the ventral cochlear nucleus to the pontine nuclei, and then to the granule cells in the cerebellum [10]. Neither the forebrain nor the thalamus is necessary for expression of the CR. Actually, the conditioned eyeblink response is successfully acquired by decerebrated animals [62]. Thus, the mechanism of CR suppression by a sleeping forebrain could not easily be interpreted as sensory gating of the pathway in the thalamus during sleep.

If the observed dependence of the CR on the hippocampal state only reflects the awake/sleep state and is irrelevant to any recognition of the context, it should appear from the first session of conditioning. However, the correlation between the hippocampal state and the CR expression was not observed at the early phase of learning but evolved over several sessions as learning proceeded (Fig. 6D), suggesting that it was acquired by learning.

Taking into consideration the previous reports by Berry and colleagues and the results of our current study, it is suggested that hippocampal activity modulates information processing over several time scales. Over the time scale of a single session, the hippocampus may modulate activity such as extracting the rule of the task or consolidating the declarative aspect of the task, while other changes occur over the time scale of a single trial. Our hypothesis is that the hippocampal formation has important roles in both the acquisition of discrimination and the expression in the asymptotic phase. Specifically, the hippocampal theta rhythm may reflect top-down attentional activity of the forebrain that modulates the dynamics of cerebellar and associated circuits on a trial-by-trial basis. We hypothesize that the appearance of state dependency in a motor reflex could be attributed to the introduction of task demand, which might require an interaction between the hippocampus and the cerebellum. The causal relationship between the hippocampal theta rhythm and the current task could be investigated in further studies by perturbation of hippocampal activity, using techniques such as the pharmacological elimination of the type 2 theta rhythm by muscarinic antagonist or external elicitation of the theta rhythm by intrahippocampal electrical excitation.

There are some possible pathways that could mediate the trial-to-trial hippocampal modulation of cerebellar activity. In other words, convergent site(s) of top-down modulation from the hippocampus and the ascending information from the CS may be involved in this modulation. Since no direct connections between the hippocampus and the cerebellum are known to exit, it is difficult to pinpoint where such a pathway may reside. One possibility is that projections from the cerebral cortex to the pontine nucleus underlie this convergence, as these are the most extensive pathways in the brain [63]. Although an intact cerebral cortex is not required for standard eyeblink conditioning [62], [64], [65], some studies report cortical involvement in eyeblink conditioning in some experimental settings [40], [66]–[69]. Even in the delay paradigm, post-conditioning perturbation of the cerebral cortex affects the expression of an acquired CR response [68]. These former studies suggest the possible involvement of the cerebral cortex in the CS-pathway in our present experiment. Another possibility is the involvement of monoaminergic modulatory systems. The cerebellum is innervated by modulatory noradrenergic (locus coeruleus) and serotonergic (raphe nucleus) systems [63], which are associated with learning of eyeblink conditioning and affect learning [70]–[72]. Further studies are needed to elucidate the pathways of top-down modulation.

In conclusion, we have shown that the rats were able to acquire a conditional discrimination task in eyeblink conditioning. Furthermore, we found that hippocampal theta was elicited by the presentation of the conditional stimuli and the elicitation of the successive CR was correlated with the successful elicitation of the hippocampal theta. These results suggest that there might be a strong interaction between the hippocampus and the cerebellum that enables flexible sensorimotor integration under the demands of behavioral contextual discrimination.

## Supporting Information

Figure S1 **Number of consecutive trials with and without CR expression.** (A) Alternation between the trial with CR (CR trial, upper plots) and without CR (no-CR trial, lower plots) during a typical session with good discrimination. All the paired trials (crosses) and CS-alone trials (closed circles) are plotted, except for the invalid trials described in the text. Shown are the data from session 7 (or the 7th session) of one rat. (B) The average number of the consecutive trials with CR (left) or without CR (right) across all the rats. For example, if a sequence of 12 trials are represented as (y n n i y n i y n n n y), where “y” denotes a trial with CR, “n” denotes a trial without CR, and “i” denotes an invalid trial, the numbers of consecutive trials without CR are (2, 1, 3), with the average 2. The average number of consecutive trials with CR response reached 2.00 in session 10, with the standard error of 0.14. The average number of consecutive trials without CR response decreased to 2.83 in session 10, with the standard error of 0.26. Assuming that CR is not elicited in the sleeping state, the average length of sleep is bounded from above by (2.83+1) trials ×35 s = 134 s. Assuming that no-CR trials indicate sleeping states, the average length of the awake period is bounded from above by (2.00+1)×35 = 105 s.
(TIF)

Figure S2 **Correlation between the pre-CS relative theta power and CR expression recalculated after confining the data to trials occurring immediately after trials showing CRs.** An analysis was conducted equivalent to that in Figure 5C, except that the data were confined to trials that immediately followed a CR-expressing awake trial to eliminate trials where the rat may have been asleep. (A) The relative theta power in the paired trials for sessions 6–10. The data in sessions 6–10 were combined, and averaged for each rat. Then, the average across the 5 rats was compared. A significant difference was observed (paired *t*-test, *P*<0.01, *n* = 5). (B) The relative theta power in the CS-alone trials for session 6–10. The data in sessions 6–10 were combined, and averaged for each rat. A significant difference was observed (paired *t*-test, *P*<0.05, *n* = 5).
(TIF)
