# Supplementary figures and images for: Hippocampal State-Dependent Behavioral Reflex to an Identical Sensory Input in Rats
Source: PLoS One. 2014 Nov 14;9(11):e112927. doi: 10.1371/journal.pone.0112927 (PMC4232594; doi:10.1371/journal.pone.0112927)

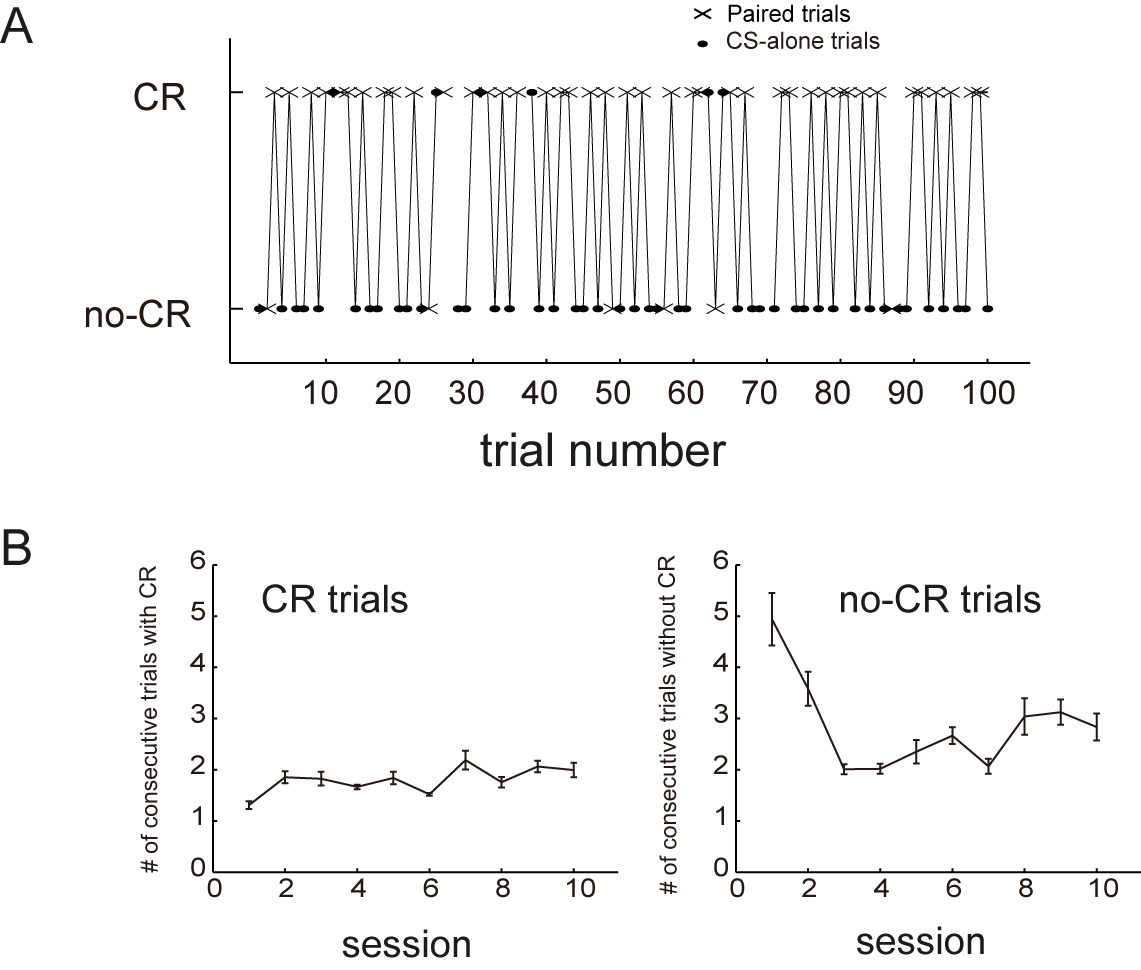

Supplement: Figure S1 — Number of consecutive trials with and without CR expression. (A) Alternation between the trial with CR (CR trial, upper plots) and without CR (no-CR trial, lower plots) during a typical session with good discrimination. All the paired trials (crosses) and CS-alone trials (closed circles) are plotted, except for the invalid trials described in the text. Shown are the data from session 7 (or the 7th session) of one rat. (B) The average number of the consecutive trials with CR (left) or without CR (right) across all the rats. For example, if a sequence of 12 trials are represented as (y n n i y n i y n n n y), where “y” denotes a trial with CR, “n” denotes a trial without CR, and “i” denotes an invalid trial, the numbers of consecutive trials without CR are (2, 1, 3), with the average 2. The average number of consecutive trials with CR response reached 2.00 in session 10, with the standard error of 0.14. The average number of consecutive trials without CR response decreased to 2.83 in session 10, with the standard error of 0.26. Assuming that CR is not elicited in the sleeping state, the average length of sleep is bounded from above by (2.83+1) trials ×35 s = 134 s. Assuming that no-CR trials indicate sleeping states, the average length of the awake period is bounded from above by (2.00+1)×35 = 105 s. (TIF) [file pone.0112927.s001.tif]

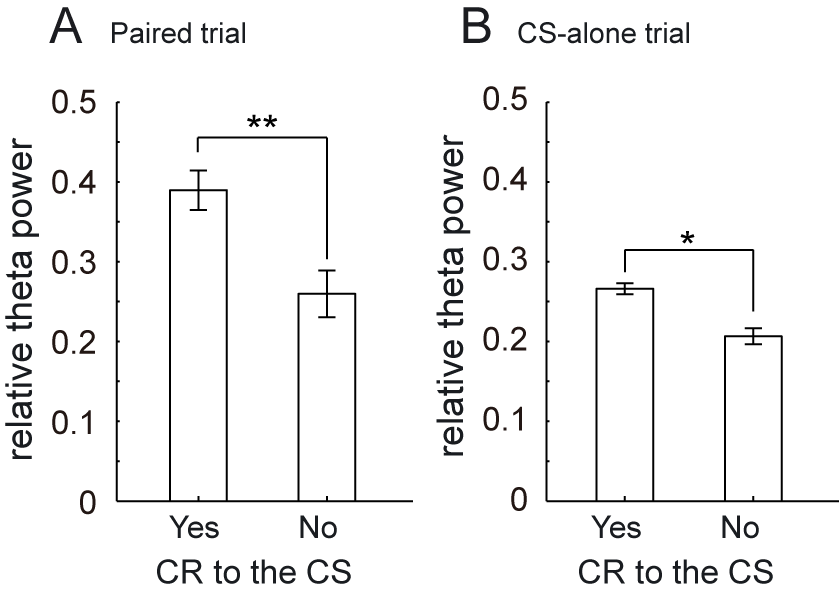

Supplement: Figure S2 — Correlation between the pre-CS relative theta power and CR expression recalculated after confining the data to trials occurring immediately after trials showing CRs. An analysis was conducted equivalent to that in Figure 5C, except that the data were confined to trials that immediately followed a CR-expressing awake trial to eliminate trials where the rat may have been asleep. (A) The relative theta power in the paired trials for sessions 6–10. The data in sessions 6–10 were combined, and averaged for each rat. Then, the average across the 5 rats was compared. A significant difference was observed (paired t-test, P<0.01, n = 5). (B) The relative theta power in the CS-alone trials for session 6–10. The data in sessions 6–10 were combined, and averaged for each rat. A significant difference was observed (paired t-test, P<0.05, n = 5). (TIF) [file pone.0112927.s002.tif]
